# Patient-Reported Outcomes After Aptos Thread-Centered Treatment in Real-World Aesthetic Practice

**DOI:** 10.7759/cureus.117245

**Published:** 2026-09-30

**Authors:** Vanessa Penteado, Felipe Penteado Marquezine, Beatriz Domenici de Oliveira, Renata Viana

**Affiliations:** 1 Aesthetic Medicine, Independent Research, São Paulo, BRA; 2 Biomedical Sciences, Independent Research, São Paulo, BRA

**Keywords:** absorbable sutures, aptos threads, facial rejuvenation, minimally invasive aesthetic procedures, patient-reported outcomes, patient satisfaction, real-world evidence, thread lift

## Abstract

Suspension thread procedures are increasingly used for minimally invasive facial rejuvenation, but real-world patient-reported outcome data remain limited. This retrospective cross-sectional survey evaluated patient-reported satisfaction, perceived aesthetic improvement, and willingness to repeat or recommend treatment following Aptos thread-centered treatment in a private aesthetic practice. Adult patients treated between 2024 and 2026 were invited to complete a digital study-specific questionnaire. Of 159 invited patients, 111 responded, and 97 provided complete numeric responses to all 0-10 scale items and constituted the analytical sample. Associated facial aesthetic procedures were documented in 25.8% of patients, including energy-based treatments in 12.4%. Outcomes included overall satisfaction, perceived improvement in skin firmness and facial contour, patient-perceived improvement in self-esteem, overall perceived aesthetic improvement, willingness to recommend the procedure, and willingness to repeat treatment. Mean overall satisfaction was 8.68 ± 1.92, with a median score of 10; 79 patients (81.4%) reported satisfaction ≥8/10, and 51 (52.6%) assigned the maximum score. Perceived aesthetic improvement was rated as “significant” or “very significant” by 72 patients (74.2%). Willingness to recommend the procedure was reported by 90 patients (92.8%), and willingness to undergo treatment again by 78 patients (80.4%). Respondents reported favorable perceptions of their treatment experience. Future prospective studies should use validated outcome measures, standardized follow-up, and structured safety assessment.

## Introduction

Nonsurgical aesthetic procedures now represent a substantial component of global aesthetic practice, reflecting increasing demand for less invasive, individualized interventions designed to provide natural-looking improvement with reduced downtime relative to surgical approaches [1,2]. Within this context, suspension thread techniques are increasingly used for soft tissue repositioning and facial rejuvenation, either as a standalone procedure or as part of combined minimally invasive treatment strategies [3-5].

Aptos threads were originally described by Sulamanidze et al. in the early 2000s as barbed sutures designed for facial soft tissue repositioning [6]. Since then, thread-lifting systems have evolved to include different configurations and absorbable materials, including poly(L-lactic acid-co-ε-caprolactone) (P(LA/CL)) threads, which are used for lifting and contouring of facial regions such as the midface, mandibular area, neck, nose, and brow [7-9]. Their clinical rationale combines immediate mechanical soft-tissue repositioning with tissue-remodeling responses that have been described for P(LA/CL)-based threads, including changes in collagen, elastin, and tissue integration during absorbable thread degradation [5,10,11]. Large real-world analyses have also documented the frequent use of combination approaches in thread-lifting practice, highlighting the multimodal nature of facial rejuvenation in routine clinical settings [12].

In aesthetic medicine, patient-reported outcomes (PROs) are particularly relevant because they capture satisfaction, self-perception, and treatment benefit as perceived by the patient, dimensions that complement objective clinical assessment and reflect outcomes that are meaningful from the patient’s perspective [13]. This perspective is particularly relevant in real-world aesthetic practice, where treatment plans are frequently individualized and may combine thread procedures with energy-based devices or other minimally invasive aesthetic interventions [14,15].

Although clinical studies have reported favorable outcomes after absorbable thread-lifting procedures, patient-reported data from routine aesthetic practice remain limited, particularly in settings where treatment plans are individualized and may include adjunctive aesthetic technologies. Capturing satisfaction, perceived improvement, and future treatment intention may provide clinically relevant insight into the patient-perceived value of thread-centered rejuvenation strategies. The primary objective of this retrospective cross-sectional survey was to describe patient-reported satisfaction, perceived aesthetic improvement, and future treatment intention following Aptos thread-centered treatment in a single private aesthetic practice. Secondary exploratory objectives were to assess associations between overall satisfaction, domain-specific patient-reported ratings, future treatment intentions, and patient age.

## Materials and methods

Study design and setting

This was a retrospective cross-sectional patient-reported satisfaction survey conducted at a private aesthetic medicine clinic in the state of São Paulo, Brazil. All thread procedures included in this survey were performed by a single physician over the period from 2024 to 2026.

Participants and data collection

The study population comprised all consecutive adult patients who had undergone at least one Aptos thread procedure at the clinic and were identified from the clinic’s electronic medical records. Eligible patients were invited to complete the questionnaire via a digital messaging application. Data were collected between March and April 2026. No additional exclusion criteria were applied. Patients were not excluded on the basis of complications, treatment dissatisfaction, or other clinical outcomes.

Questionnaire responses were initially linked to clinic treatment records for verification of procedural information. Following verification, all direct patient identifiers, including names and contact details, were removed from the analytical dataset. The anonymized dataset used for all analyses reported herein contains no information capable of directly identifying individual participants.

Questionnaire and outcomes

A study-specific structured questionnaire was developed for quality-of-care assessment purposes to evaluate patient-reported satisfaction, perceived aesthetic improvement, and future treatment intention after thread procedures. The questionnaire was self-completed by participants and focused on outcomes based on the patient’s own perception of the treatment result. These outcomes represented retrospective global ratings rather than change scores derived from baseline and follow-up assessments.

The questionnaire was developed by the authors specifically for this study, based on their clinical experience with thread-centered aesthetic treatments. No formal literature review, patient interviews, cognitive debriefing, or pilot testing was conducted.

PROs included overall satisfaction with the procedure result, rated on a 0-10 numeric rating scale, and perceived improvement in the treated area. No predefined verbal anchors or definitions were provided for the numeric scales or categorical improvement levels. Participants rated perceived improvement in skin firmness and facial contour and patient-perceived improvement in self-esteem, each using a single non-validated 0-10 item. In addition, participants classified their overall perceived aesthetic improvement in the treated region as very significant, significant, moderate, mild, or absent.

Future treatment intention was assessed through questions regarding interest in treating additional anatomical regions with threads, willingness to undergo the procedure again, and willingness to recommend the procedure to another person. Response options for these items were “yes,” “maybe,” or “no.” These items measured stated intentions only and did not assess subsequent treatment behavior or scheduled repeat procedures. The PRO items included in the analysis, with their original Portuguese wording and English translation, are provided in Appendix A. Treatment indication and suitability for thread lifting were determined by the treating physician based on clinical facial assessment and alignment of patient expectations. The questionnaire was study-specific, non-validated, and did not reproduce any copyrighted or licensed instrument. The selection of PRO domains was informed by published approaches to PRO assessment in facial aesthetic research [13].

The timing and continuation of associated procedures relative to thread placement were not systematically assessed. Only respondents who provided complete numeric responses to all 0-10 scale items were included in the analytical sample to maintain a consistent sample across outcomes.

Treatment characteristics

Procedural information was extracted from the clinic’s treatment records and included the date of each thread procedure, number of thread treatment sessions, and associated facial aesthetic procedures. The clinical indication for additional thread sessions was not systematically classified; therefore, these sessions should not be interpreted as repeat procedures after loss of effect. Associated procedures were categorized as botulinum toxin, energy-based treatment, hyaluronic acid filler, or polydioxanone (PDO) threads. The timing of associated procedures relative to thread placement was not systematically abstracted, and no predefined periprocedural time window was applied. The absence of an associated procedure in the clinical record was considered to indicate that no additional facial aesthetic procedure had been performed. Procedures not clearly related to facial aesthetic treatment were excluded from this classification.

For patients who underwent more than one thread treatment session, the first recorded thread procedure was defined as the index procedure. The follow-up interval was calculated from the index procedure to questionnaire completion and categorized as <3 months, 3 to <6 months, 6 to <12 months, 12 to <18 months, or ≥18 months. All procedures involved commercially available Aptos threads from the Visage Excellence and Light Lift lines.

Statistical analysis

Continuous variables are reported as mean ± SD and, where appropriate, median and range. Categorical variables are presented as absolute frequencies and percentages. The threshold of ≥8/10 was selected solely to facilitate the descriptive presentation of overall satisfaction and was not intended as a validated clinical cutoff. Outcomes were initially analyzed for the complete analytical sample and subsequently stratified by follow-up interval (<3 months, 3 to <6 months, 6 to <12 months, 12 to <18 months, and ≥18 months). No inferential comparisons between follow-up intervals were performed. Subgroup estimates were considered descriptive and should not be interpreted as precise estimates because of the small denominators. Wilson score 95% CIs were calculated for key proportions. For ordinal analyses, perceived improvement was coded as 0 = no improvement, 1 = mild, 2 = moderate, 3 = significant, and 4 = very significant; future-intention responses were coded as 0 = no, 1 = maybe, and 2 = yes. Spearman’s rank correlation coefficient (ρ) was used to assess associations involving ordinal variables and the association between time from the index procedure to survey completion and overall satisfaction. Approximate 95% CIs for Spearman’s ρ were calculated using Fisher’s z transformation. All analyses were exploratory and hypothesis-generating; no correction for multiple comparisons was applied. A p-value < 0.05 was considered nominally significant. Statistical analyses were performed using Microsoft Excel (Microsoft Corporation, Redmond, WA, USA). Claude Sonnet 4.6 (Anthropic, PBC, San Francisco, CA, USA) was used to cross-check the statistical results and assist with figure preparation. The authors verified the spreadsheet formulas and checked the reported counts, percentages, correlations, tables, and figures against the underlying dataset.

Ethical considerations

This study involved the retrospective analysis of de-identified patient satisfaction survey data and clinical record information collected as part of routine quality-of-care assessment activities. Participation in the satisfaction survey was voluntary, and all direct identifiers were removed before analysis. Patients had previously authorized the use of de-identified clinical data for quality-of-care assessment and scientific purposes as part of the clinic’s standard documentation procedures. No patient images or direct patient identifiers are included in the manuscript or analytical dataset. The study was conducted in accordance with the principles of the Declaration of Helsinki.

## Results

Patient and treatment characteristics

Of 159 patients invited to participate, 111 responded (response rate, 69.8%). After excluding 14 respondents who did not provide complete numeric responses to all 0-10 scale items, 97 patients constituted the analytical sample (Figure 1).

**Figure 1:**
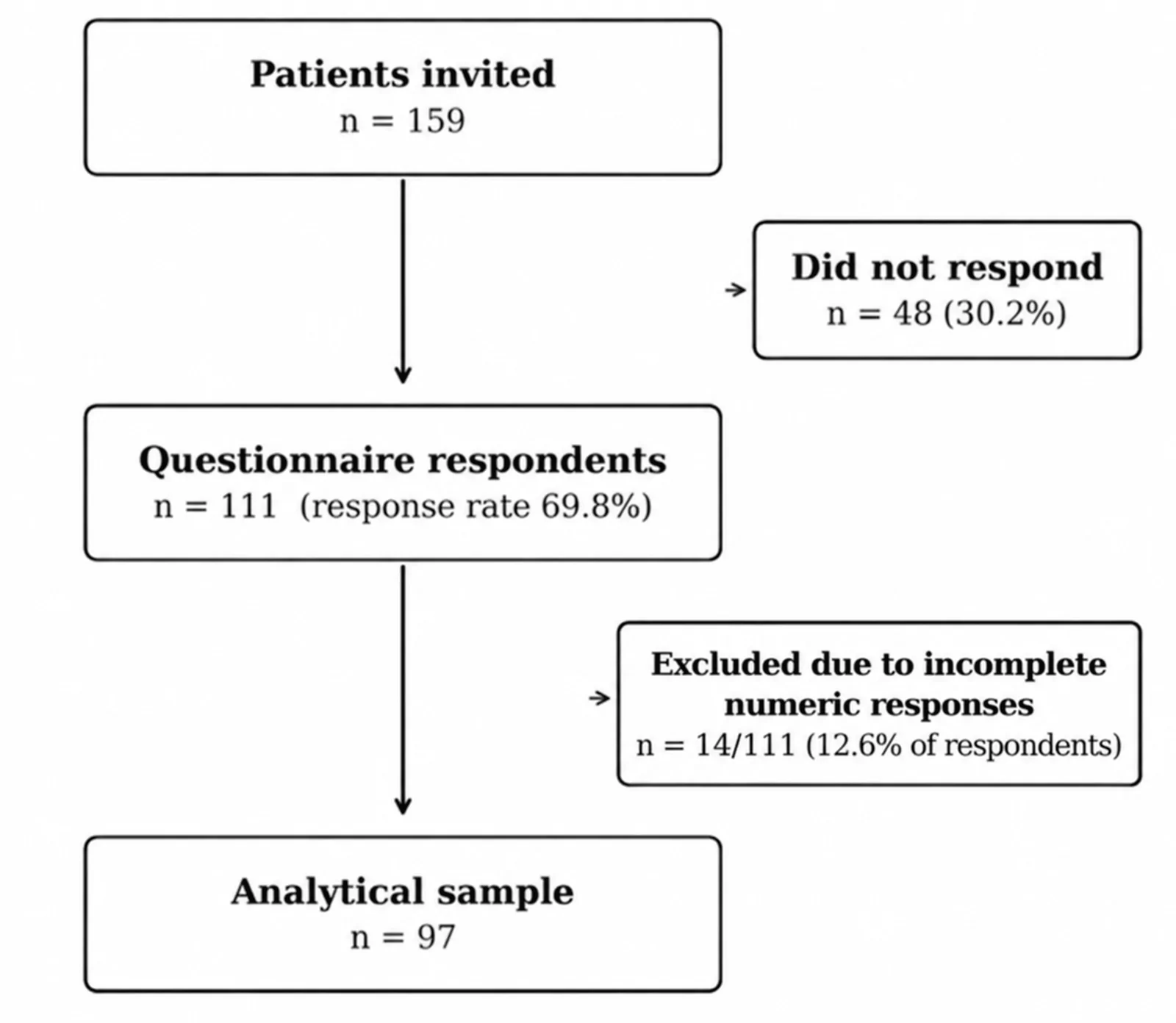
Participant flow diagram.

Patient and treatment characteristics are summarized in Table 1. The mean age was 50.6 ± 10.2 years. Most patients were female (n = 92; 94.8%), while five were male (5.2%). Overall, 87 patients (89.7%) underwent one thread treatment session, nine (9.3%) underwent two sessions, and one (1.0%) underwent three sessions. At least one associated facial aesthetic procedure was recorded for 25 patients (25.8%), including botulinum toxin in 15 (15.5%), energy-based treatment in 12 (12.4%), hyaluronic acid filler in five (5.2%), and PDO threads in one (1.0%). The median follow-up interval was 7.1 months (IQR, 4.8-13.6 months; range, 0.2-24.1 months).

**Table 1:** Patient and treatment characteristics (n = 97). The index procedure was defined as the first recorded thread treatment. ^†^ More than one associated facial aesthetic procedure could be recorded per patient; percentages were calculated using the total sample (n = 97) and may not sum to 100%. PDO, polydioxanone

Characteristic	Value
Age, years	
Mean ± SD	50.6 ± 10.2
Sex, n (%)	
Female	92 (94.8)
Male	5 (5.2)
Follow-up interval from index procedure to survey, n (%)	
<3 months	9 (9.3)
3 to <6 months	33 (34.0)
6 to <12 months	26 (26.8)
12 to <18 months	22 (22.7)
≥18 months	7 (7.2)
Number of treatment sessions, n (%)	
One session	87 (89.7)
Two sessions	9 (9.3)
Three sessions	1 (1.0)
Treatment approach, n (%)	
Thread treatment without additional facial aesthetic procedure recorded	72 (74.2)
Thread treatment with ≥1 associated facial aesthetic procedure	25 (25.8)
Associated treatment type, n (%)^†^	
Botulinum toxin	15 (15.5)
Energy-based treatment	12 (12.4)
Hyaluronic acid filler	5 (5.2)
PDO threads	1 (1.0)

Overall patient-reported satisfaction

Overall satisfaction scores are presented in Figure 2. The median overall satisfaction score was 10 (IQR, 8-10; range, 0-10), and the mean was 8.68 ± 1.92 (n = 97). The distribution was skewed toward higher scores, with 79 patients (81.4%; 95% CI, 72.6%-87.9%) reporting satisfaction ≥8/10 and 51 patients (52.6%; 95% CI, 42.7%-62.2%) reporting the maximum score of 10/10 (Figure 2A). When descriptively stratified according to the follow-up interval from the index procedure to survey completion, mean satisfaction scores were 9.1 at <3 months (n = 9), 8.5 at 3 to <6 months (n = 33), 8.7 at 6 to <12 months (n = 26), 8.7 at 12 to <18 months (n = 22), and 8.7 at ≥18 months (n = 7) (Figure 2B).

**Figure 2:**
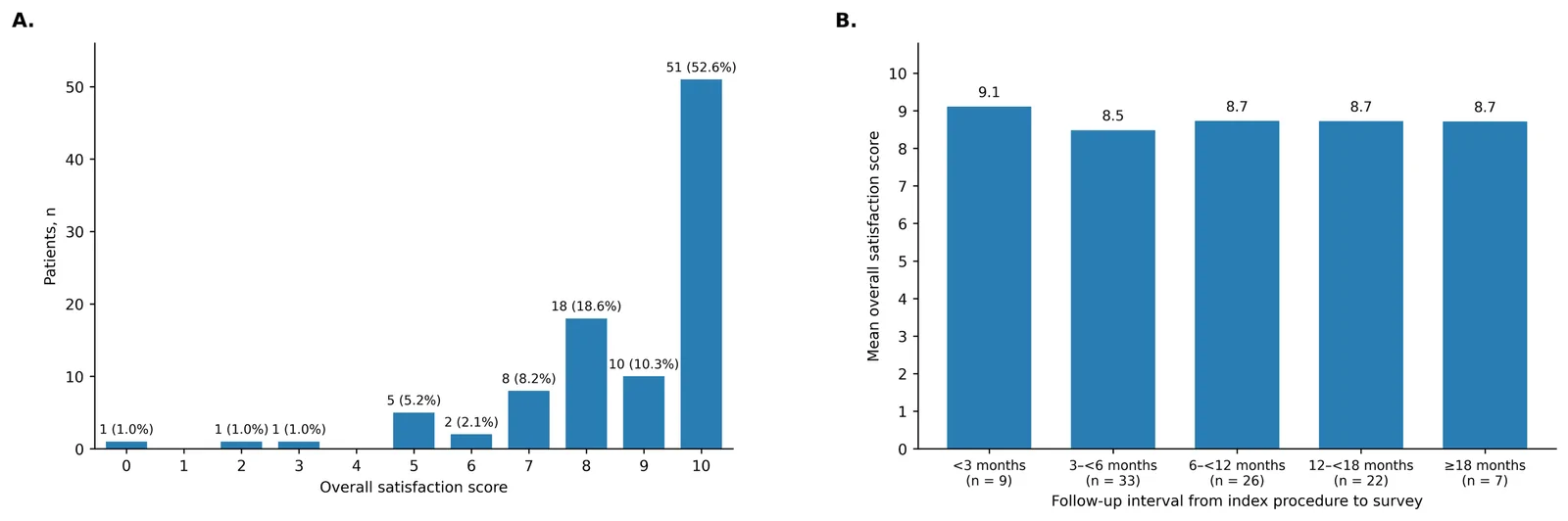
Overall satisfaction after thread procedures. (A) Distribution of overall satisfaction scores in the total sample; values are presented as n (%), and categories with no responses are displayed without data labels. (B) Mean overall satisfaction descriptively stratified according to the follow-up interval from the index procedure to survey completion.

Domain-specific patient-reported scores

Domain-specific scores are summarized in Table 2. In the total sample, the mean scores were 8.4 ± 2.0 for skin firmness, 8.6 ± 2.0 for facial contour, and 8.8 ± 2.0 for patient-perceived improvement in self-esteem. Descriptive stratification according to the follow-up interval from the index procedure to survey completion showed high mean scores across all three domains and time categories.

**Table 2:** Domain-specific patient-reported scores.

Domain	Total sample (n = 97)	<3 months (n = 9)	3 to <6 months (n = 33)	6 to <12 months (n = 26)	12 to <18 months (n = 22)	≥18 months (n = 7)
Skin firmness score	8.4 ± 2.0	8.9 ± 1.3	8.1 ± 2.2	8.4 ± 1.8	8.6 ± 2.4	8.6 ± 1.1
Facial contour	8.6 ± 2.0	8.9 ± 1.3	8.4 ± 2.2	8.4 ± 1.9	8.7 ± 2.4	9.0 ± 1.0
Patient-perceived improvement in self-esteem	8.8 ± 2.0	9.2 ± 1.3	8.5 ± 2.2	8.8 ± 1.8	8.7 ± 2.4	9.4 ± 1.1

Perceived aesthetic improvement

Overall, 72 patients (74.2%; 95% CI, 64.7%-81.9%) reported “significant” or “very significant” perceived aesthetic improvement. Specifically, 44 patients (45.4%) rated their improvement as very significant, and 28 patients (28.9%) rated it as significant. Moderate improvement was reported by 16 patients (16.5%), mild improvement by seven patients (7.2%), and no perceived improvement by two patients (2.1%) (Figure 3A). When descriptively stratified according to the follow-up interval from the index procedure to survey completion, “significant” or “very significant” improvement was reported by 88.9% of patients at <3 months, 69.7% at 3 to <6 months, 73.1% at 6 to <12 months, 72.7% at 12 to <18 months, and 85.7% at ≥18 months (Figure 3B).

**Figure 3:**
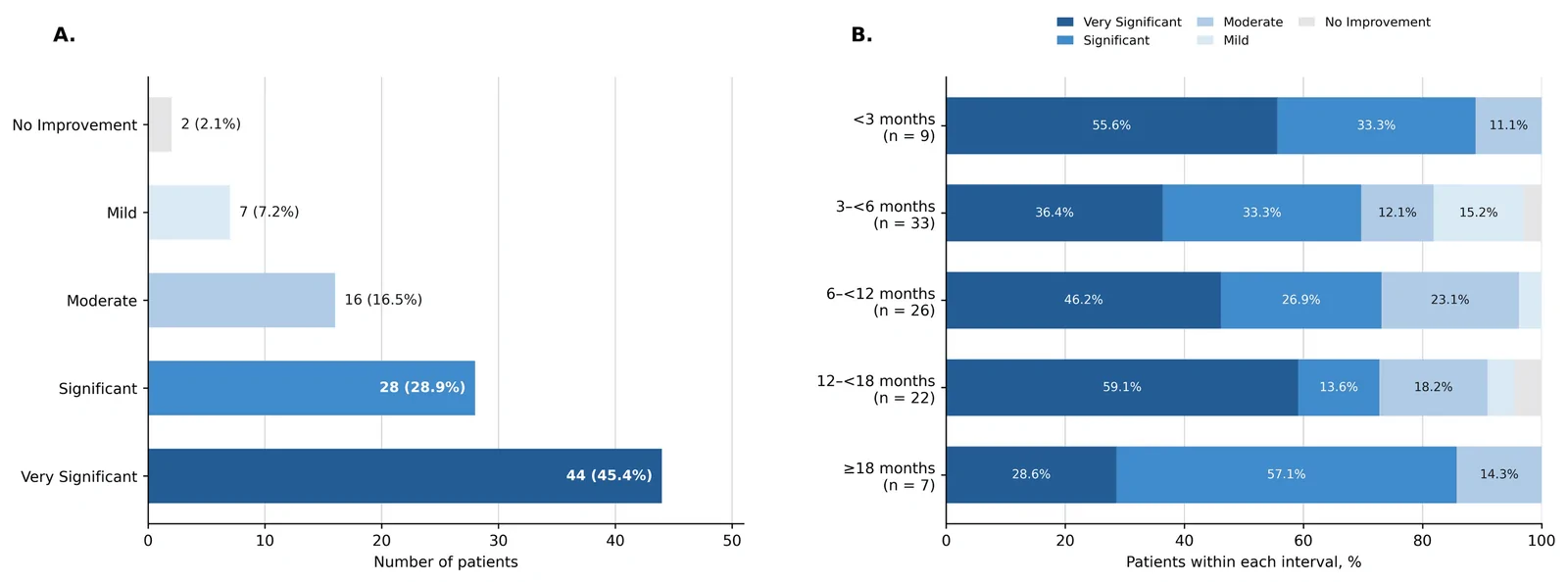
Perceived aesthetic improvement after thread procedures. (A) Distribution of perceived aesthetic improvement in the total sample; values are presented as n (%). (B) Distribution of perceived aesthetic improvement descriptively stratified according to the follow-up interval from the index procedure to survey completion; percentages were calculated within each interval.

Future treatment intention and recommendation

Future treatment intention and willingness to recommend the procedure are illustrated in Figure 4. Willingness to recommend the procedure was reported by 90 patients (92.8%), whereas seven patients (7.2%) would not recommend it and zero patients (0.0%) selected “maybe.” Willingness to undergo the procedure again was reported by 78 patients (80.4%), while 11 (11.3%) were undecided and eight (8.2%) would not repeat the treatment. Interest in treating additional anatomical areas was expressed by 66 patients (68.0%), with 20 patients (20.6%) undecided and 11 patients (11.3%) not interested.

**Figure 4:**
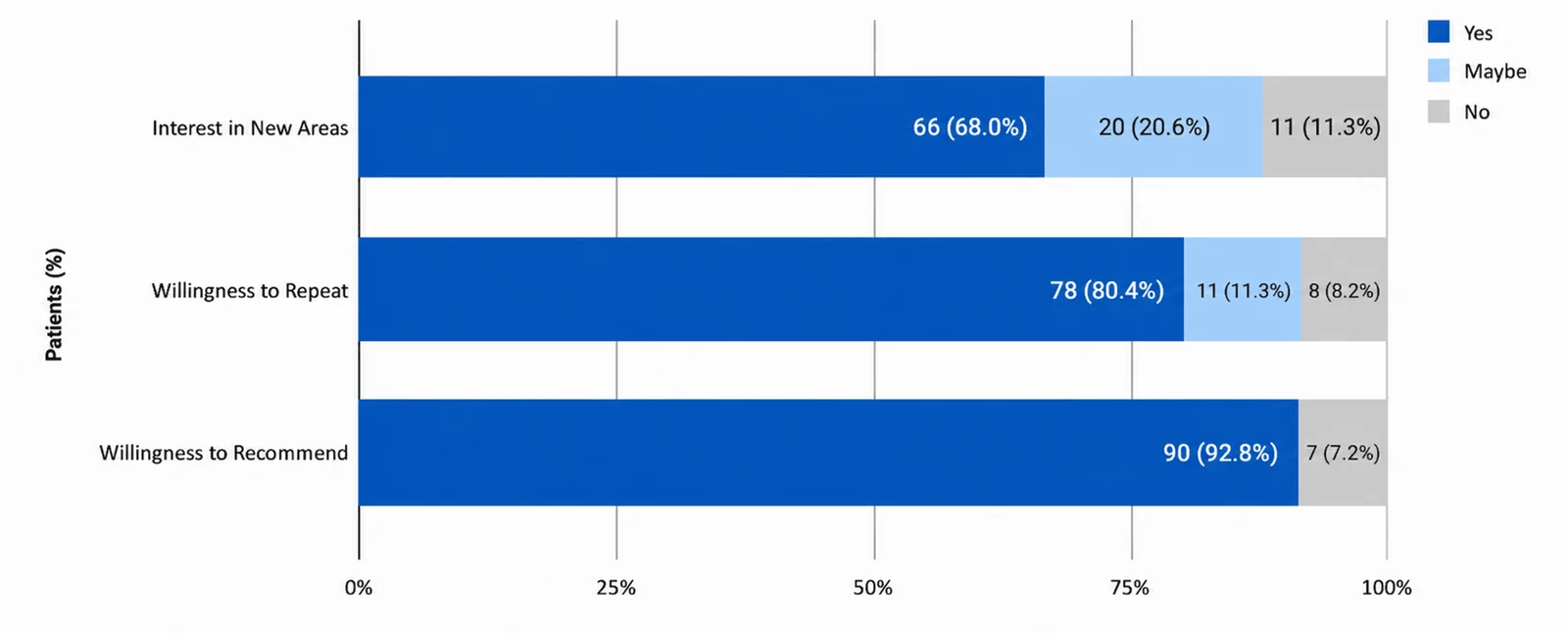
Future treatment intention and willingness to recommend the procedure. Values are presented as n (%).

Exploratory correlation analyses

Correlation Between Overall Satisfaction and Domain-Specific Subscores

Spearman’s rank correlation analyses revealed strong positive associations between overall satisfaction and the selected PRO variables (Table 3). The strongest correlation was observed between overall satisfaction and facial contour score (ρ = 0.895, p < 0.001), followed by perceived aesthetic improvement (ρ = 0.843, p < 0.001), patient-perceived improvement in self-esteem (ρ = 0.839, p < 0.001), and skin firmness score (ρ = 0.825, p < 0.001; all n = 97).

**Table 3:** Spearman rank correlations between overall satisfaction score and selected PRO variables (n = 97). All correlations are two-tailed Spearman’s ρ. Approximate 95% CIs were calculated using Fisher’s z transformation. PRO, patient-reported outcome

PRO variable	Spearman’s ρ	95% CI for ρ	p-value	n
Facial contour score	0.895	0.847-0.929	<0.001	97
Perceived aesthetic improvement	0.843	0.774-0.892	<0.001	97
Patient-perceived improvement in self-esteem	0.839	0.768-0.890	<0.001	97
Skin firmness score	0.825	0.749-0.880	<0.001	97

Association Between Perceived Aesthetic Improvement and Future Treatment Intention

Future treatment intention outcomes varied across perceived improvement categories (Table 4). These subgroup percentages are descriptive and should be interpreted cautiously because of the small denominators, particularly in the mild- and no-improvement categories. Among patients who rated their improvement as very significant or significant, willingness to recommend was reported by 100% in both categories, and willingness to repeat the procedure was reported by 97.7% and 92.9%, respectively. Neither of the two patients who reported no perceived improvement expressed willingness to recommend or repeat the procedure.

**Table 4:** Future treatment intention outcomes stratified by perceived aesthetic improvement category (n = 97).

Perceived improvement category	n	Willingness to recommend, n (%)	Willingness to repeat, n (%)
Very significant	44	44 (100%)	43 (97.7%)
Significant	28	28 (100%)	26 (92.9%)
Moderate	16	12 (75.0%)	7 (43.8%)
Mild	7	6 (85.7%)	2 (28.6%)
No perceived improvement	2	0 (0%)	0 (0%)

Spearman’s correlation showed that perceived improvement was significantly associated with willingness to repeat the procedure (ρ = 0.589; 95% CI, 0.441-0.706; p < 0.001) and willingness to recommend it (ρ = 0.401; 95% CI, 0.219-0.556; p < 0.001). Overall satisfaction score was also significantly associated with willingness to repeat the procedure (ρ = 0.630; 95% CI, 0.492-0.737; p < 0.001).

Association Between Patient Age and Overall Satisfaction

A weak negative unadjusted correlation was observed between patient age and overall satisfaction score (Spearman’s ρ = -0.261; 95% CI, -0.438 to -0.065; p = 0.010; n = 97). This association was exploratory and was not adjusted for potential confounding variables.

Association Between Time Since the Index Procedure and Overall Satisfaction

Overall satisfaction was consistently high across the follow-up intervals, with mean scores of 9.1 at <3 months, 8.5 at 3 to <6 months, 8.7 at 6 to <12 months, 8.7 at 12 to <18 months, and 8.7 at ≥18 months (Figure 2B). Consistent with this descriptive pattern, the time from the index procedure to survey completion was not significantly associated with overall satisfaction (Spearman’s ρ = -0.059; 95% CI, -0.255 to 0.142; p = 0.569; n = 97). These findings indicate that overall satisfaction did not show evidence of a decrease with increasing time since the procedure in this cross-sectional sample.

## Discussion

This cross-sectional real-world survey found high patient-reported satisfaction and favorable future treatment intention among 97 patients who underwent Aptos thread procedures at a single private aesthetic practice. More than 80% of patients reported overall satisfaction ≥8/10, more than half assigned the maximum satisfaction score, and nearly three-quarters perceived their aesthetic improvement as “significant” or “very significant.” In addition, willingness to recommend the procedure and willingness to undergo treatment again were high, suggesting a favorable patient-perceived value of thread-centered facial rejuvenation in routine clinical practice.

Previous thread-lifting studies provide contextual evidence regarding patient satisfaction and perceived aesthetic improvement, although they are not directly comparable with the present study. In a prospective interventional study using Aptos P(LA/CL)-hyaluronic acid threads, Al-Anssari reported favorable clinical improvement, high patient satisfaction, and positive GAIS outcomes across different grades of facial laxity [16]. Similarly, Singh et al. reported favorable physician- and subject-rated aesthetic improvement in a multicenter real-world study of absorbable thread lifting, supporting the relevance of patient-perceived improvement in thread-lifting outcomes [17]. Differences in study design, thread product, outcome definitions, follow-up, and assessment instruments, together with the retrospective non-validated ratings used in the present study, preclude direct comparison. These studies are therefore cited only as contextual evidence.

In addition to overall satisfaction, domain-specific scores for skin firmness, facial contour, and patient-perceived improvement in self-esteem were consistently favorable and strongly correlated with overall satisfaction. However, because these closely related outcomes were collected simultaneously using the same non-validated questionnaire, the observed correlations may reflect conceptual overlap, common-method variance, and ceiling effects rather than independent confirmation of treatment benefit. In aesthetic medicine, PROs are particularly relevant because treatment success is closely related to the patient’s perception of improvement, satisfaction, and confidence. However, because the questionnaire used in this study was not a validated psychometric instrument, these findings should be interpreted as descriptive patient-reported data rather than as validated quality-of-life or satisfaction measures. Because the study-specific items were collected concurrently and assessed related aspects of the same treatment experience, the strong correlations may partly reflect shared-method variance or a global evaluation (halo) effect. Future studies should incorporate validated PRO instruments, such as FACE-Q modules, when appropriately licensed or permitted, to allow more standardized assessment and comparison across studies.

Perceived aesthetic improvement was also associated with future treatment intention. Patients who reported significant or very significant improvement were more likely to express willingness to recommend the procedure and undergo treatment again. This finding is clinically relevant because willingness to repeat or recommend treatment may reflect not only perceived efficacy but also the overall acceptability of the procedure in routine practice. Nevertheless, these associations should not be interpreted as causal. Patients who were more satisfied with their aesthetic outcome would be expected to report greater willingness to repeat or recommend the procedure, and the cross-sectional design does not allow determination of temporal or causal relationships.

A weak unadjusted correlation between patient age and overall satisfaction was identified. This association should not be attributed to age itself because potential confounding factors, including baseline laxity, treatment region, procedural characteristics, follow-up duration, and associated technologies, were not assessed in an adjusted analysis. Hügül et al. reported that patient satisfaction after thread lifting was not significantly associated with age, treatment area, thread type, or number of threads used [18]. Therefore, the age-related finding in the present study should be considered hypothesis-generating. Future prospective studies should include standardized baseline laxity assessments and expectation-related measures to better clarify the relationship between age, baseline severity, treatment indication, and patient satisfaction.

Overall satisfaction was descriptively high across the predefined follow-up intervals, and no significant association was observed between the time from the index procedure to survey completion and overall satisfaction. These findings provide no evidence of lower satisfaction among patients surveyed at longer intervals after the procedure. However, because each patient was assessed at a single time point, this cross-sectional analysis cannot establish the longitudinal maintenance or durability of treatment effects. Prospective studies with repeated assessments at standardized follow-up time points are needed to evaluate changes in satisfaction over time.

In this cohort, 25.8% of patients underwent at least one associated facial aesthetic procedure. This pattern is consistent with contemporary multimodal facial rejuvenation practice, in which thread procedures may be combined with other minimally invasive or energy-based interventions according to individual patient needs [12,14,19]. In this context, treatment confounding prevents separation of the perceived effects of threads from those of energy-based or other associated treatments. Perfeito et al. separately evaluated the safety of Aptos Excellence Visage threads combined with same-day energy-based technologies under ultrasound guidance [20]. However, those findings cannot be extrapolated to the present cohort, in which adverse events were not systematically assessed.

This study adds real-world patient-reported data on satisfaction, perceived aesthetic improvement, and future treatment intention after Aptos thread procedures in routine aesthetic practice. However, findings should be interpreted considering the retrospective cross-sectional design, absence of baseline assessment or control group, use of a non-validated questionnaire, variable interval between treatment and survey response, single-clinic/single-physician setting, and use of associated aesthetic technologies in part of the cohort. Recall bias is an additional limitation of this study, particularly among participants treated in 2024, who had a longer interval between treatment and questionnaire completion than those treated more recently. Because 48 of 159 invited patients did not respond and 14 of 111 respondents were excluded for incomplete numeric responses, nonresponse bias and selection associated with complete-case analysis cannot be excluded. Respondents may have differed systematically from nonrespondents, and more satisfied or engaged patients may have been more likely to participate, potentially inflating the favorable outcome estimates. The survey was not designed for systematic safety assessment; therefore, no conclusions regarding safety, complication frequency, tolerability, or risk-benefit balance can be drawn. The timing of co-interventions was not standardized, and their influence could not be separately assessed. Additional measurement limitations include the absence of standardized scale anchors and baseline assessment of patient expectations, as well as potential common-method variance among outcomes collected simultaneously. The pronounced ceiling effect in overall satisfaction may have limited the scale’s ability to discriminate among highly satisfied patients. Accordingly, these findings describe patient-reported experience among respondents from a single-practice treatment cohort and do not establish the efficacy or comparative value of thread-centered rejuvenation relative to alternative treatments or no treatment. Future prospective studies should include validated PRO measures, standardized follow-up, objective clinical assessment, and structured safety reporting.

## Conclusions

The 97 complete-case survey respondents reported favorable perceptions of their treatment experience. However, these findings reflect the overall treatment setting and cannot be attributed specifically to Aptos threads. Further prospective controlled studies are needed to confirm these findings.

## Appendices

Appendix A 

**Table 5:** Study questionnaire. This was a study-specific, non-validated questionnaire developed for quality-of-care assessment. Items listed separately were answered individually by each participant.

Português	English
Título: Procedimento com Fios no rosto	Title: Facial Thread Procedure
Instruções introdutórias: Estou realizando um estudo de satisfação com pacientes que realizaram fios comigo desde fevereiro de 2024. Sua opinião é muito importante para avaliarmos resultados reais e aprimorar ainda mais a prática clínica. O questionário é rápido (menos de 3 minutos) e confidencial.	Introductory instructions: I am conducting a satisfaction study involving patients who have undergone thread procedures with me since February 2024. Your opinion is very important for evaluating real-world results and further improving clinical practice. The questionnaire is brief (less than 3 minutes) and confidential.
1. Percebeu a melhoria na região tratada? Opções de resposta: Leve; Moderada; Importante; Muito importante; Não percebi melhora.	1. Did you perceive improvement in the treated region? Response options: Mild; Moderate; Significant; Very significant; I did not perceive any improvement.
2. Nota geral de satisfação (0–10) Resposta numérica aberta.	2. Overall satisfaction score (0–10) Open numeric response.
3. Melhorou a firmeza da pele? (0–10) Resposta numérica aberta.	3. Did skin firmness improve? (0–10) Open numeric response.
4. Melhorou o contorno facial? (0–10) Resposta numérica aberta.	4. Did facial contour improve? (0–10) Open numeric response.
5. Melhorou a autoestima? (0–10) Resposta numérica aberta.	5. Did self-esteem improve? (0–10) Open numeric response.
6. Tem interesse em realizar Fios em outra região? Opções de resposta: Sim; Não; Talvez.	6. Are you interested in undergoing thread treatment in another region? Response options: Yes; No; Maybe.
7. Faria novamente? Opções de resposta: Sim; Não; Talvez.	7. Would you undergo the procedure again? Response options: Yes; No; Maybe.
8. Indicaria para outra pessoa? Opções de resposta: Sim; Não; Talvez.	8. Would you recommend the procedure to another person? Response options: Yes; No; Maybe.
